# Prevalence of Integrons and Insertion Sequences in ESBL-Producing *E. coli* Isolated from Different Sources in Navarra, Spain

**DOI:** 10.3390/ijerph15102308

**Published:** 2018-10-20

**Authors:** Lara Pérez-Etayo, Melibea Berzosa, David González, Ana Isabel Vitas

**Affiliations:** 1 Department of Microbiology and Parasitology, University of Navarra, 31008 Pamplona, Spain; mberzosa.1@alumni.unav.es (M.B.); dgonzalez@unav.es (D.G.); avitas@unav.es (A.I.V.); 2 IDISNA, Navarra Health Research Institute, 31008 Pamplona, Spain

**Keywords:** ESBL-producing *E. coli*, β-lactamase genes, antimicrobial resistance, integrons, insertion sequences

## Abstract

Mobile genetic elements play an important role in the dissemination of antibiotic resistant bacteria among human and environmental sources. Therefore, the aim of this study was to determine the occurrence and patterns of integrons and insertion sequences of extended-spectrum β-lactamase (ESBL)-producing *Escherichia coli* isolated from different sources in Navarra, northern Spain. A total of 150 isolates coming from food products, farms and feeds, aquatic environments, and humans (healthy people and hospital inpatients), were analyzed. PCRs were applied for the study of class 1, 2, and 3 integrons (*intI1*, *intI2*, and *intI3*), as well as for the determination of insertion sequences (IS*26*, IS*Ecp1*, IS*CR1*, and IS*903*). Results show the wide presence and dissemination of *intI1* (92%), while *intI3* was not detected. It is remarkable, the prevalence of *intI2* among food isolates, as well as the co-existence of class 1 and class 2 (8% of isolates). The majority of isolates have two or three IS elements, with the most common being IS*26* (99.4%). The genetic pattern IS*26*–IS*Ecp1* (related with the pathogen clone ST131) was present in the 22% of isolates (including human isolates). In addition, the combination IS*Ecp1*–IS*26*–IS*903*–IS*CR1* was detected in 11 isolates being, to our knowledge, the first study that describes this genetic complex. Due to the wide variability observed, no relationship was determined among these mobile genetic elements and β-lactam resistance. More investigations regarding the genetic composition of these elements are needed to understand the role of multiple types of integrons and insertion sequences on the dissemination of antimicrobial resistance genes among different environments.

## 1. Introduction

Antimicrobial resistance (AMR) has become a public health problem, reaching alarming levels in many parts of the world [1,2]. In recent years, resistances in the Enterobacteriaceae family have increased significantly because of the extensive use of antibiotics in human treatment, veterinary, and agriculture, leading to the selection and global spread of resistant clones [3,4]. In particular, the dissemination of extended-spectrum β-lactamases (ESBLs) have increased dramatically in the recent years, becoming a serious global threat [5,6].

Several genetic mechanisms have been involved in the acquisition and dispersion of antimicrobial resistances. The commonly called “mobilome” [7,8] is composed of a variety of mobile genetic elements (MGEs), including plasmids, transposons (Tn), insertion sequences (IS), integrons (*intI*), and introns. Conjugation, transformation, and transduction are the main mechanisms for the horizontal transfer of MGEs [9,10].

Integrons are DNA elements capable of capturing gene cassettes (including antimicrobial resistance genes) and disseminating them using an MGE [11]. Integrons are usually composed of two conserved segments (termed 5′-conserved region (5′-CS) and 3′-conserved region (3′-CS)) separated by a variable region which contains the gene cassettes. The 5′-CS end includes (i) the *int* gene coding for an integrase, that belongs to a distinct family of the tyrosine-recombinase; (ii) a primary recombination site (*attI*); and (iii) a promoter (Pc), which ensures the transcription of the cassette genes. On the other hand, the 3′-CS region is formed by (i) a truncated gene of resistance to quaternary ammonium compounds (*qacEΔ1*); (ii) a sulfonamide resistance gene (*sul1*); and (iii) an unknown function sequence (orf5) [12]. Class 1 (*intI1*) and class 2 (*intI2*) integrons are the most commonly involved in antibiotic resistances [13,14,15,16,17], while limited work has shown the presence of class 3 (*intI3*) in Enterobacteriaceae. The gene *intI3* was reported for the first time in a carbapenem-resistant *Serratia marcescens* strain [18] and has been also detected in *Klebsiella pneumoniae* isolates [19] and other Enterobacteriaceae [20]. In addition, *bla* ESBL genes have been associated with insertion sequences. These IS are the smallest transposable elements (<2.5 kb), and are classified into families according to different characteristics, with transposases (enzymes that catalyze the IS movement) being the main classification system used [21,22]. It has been well documented that IS*26*, IS*Ecp1*, IS*CR1*, and IS*903*, in association with class 1 integrons, are the most involved elements in the antimicrobial resistance to β-lactamics [23,24,25,26,27].

Therefore, the investigation of these elements might be critical, in order to predict the potential spread of ESBL-producing strains. In this context, the aim of this study was to evaluate the presence of different types of integrons (*intI1*, *intI2*, and *intI3*) and insertion sequences (IS*Ecp1*, IS*26*, IS*CR1*, and IS*903*) in a collection of 150 ESBL-producing *E. coli* isolated from different sources in Navarra, Spain.

## 2. Materials and Methods

### 2.1. Sample Collection

A total of 150 ESBL-producing *Escherichia coli* were selected from a wide collection of ESBL-producing Enterobacteriaceae, isolated in Navarra from different environments: food products (*n* = 48), farms and feeds (*n* = 20), rivers and wastewater treatment plants (WWTPs) (*n* = 33) and human origins, including healthy volunteers (*n* = 13) and hospital inpatients (*n* = 36). Clinical isolates from hospital inpatients were provided by Clínica Universidad de Navarra, and were collected from January 2009 to December 2012 [5]. Food and environmental samples were collected from different locations in Navarra in two sampling periods (2010–2013 [5,28,29]; 2015–2016 [30]) and, finally, isolates from healthy people were collected from September 2015 to September 2016 (data not published). All samples were already identified, and phenotypically and genotypically characterized, in order to know the antimicrobial susceptibility pattern, the types of β-lactamase genes, and the phylogenetic group [28,29,30]. Isolates were selected according to the following criteria: they must show multidrug-resistant phenotype (to at least three different classes of antimicrobials) and must carry at least one ESBL gene. The main characteristics of the selected isolates, regarding type of ESBL, is shown in Table 1. Resistance profiles and complete information of each isolate is presented in the Supplementary Material, Tables S1–S6).

### 2.2. DNA Extraction and Detection of Integrons

DNA extraction was performed with DNeasy^®^ Blood & Tissue kit (Qiagen, Barcelona, Spain), using a pre-treatment protocol for Gram-negative bacteria, and following the manufacturer’s instruction. The quantity and quality of the DNA was analyzed using a Nanodrop ND-1000 spectrophotometer (NanoDrop Technologies, Wilmington, DE, USA).

Detection of class 1, class 2, and class 3 integrons in ESBL-producing *E. coli* was performed according to PCR, as described by Mazel et al. [31], and using only the primers shown in Table 2.

DNA amplification was performed in a DNA thermal cycler GeneAmp^®^ PCR system 2700 (Applied Biosystems Division, Foster City, CA, USA) in a final volume of 25 µL containing 2 µL of DNA extract mixed with 2.5 µL of 10× buffer (Bioline, London, UK), 5 µL of dNTPs (Bioline, London, UK), 1.5 µL of MgCl_2_ 50 mM (Bioline, London, UK), 2 µL of each primer Sigma-Aldrich, Steinheim, Germany), and 1.5 U of Inmolase™ DNA polymerase (Bioline, London, UK). The conditions of the amplification were as follows: initial denaturation at 94 °C for 10 min, followed by 30 cycles of DNA denaturation at 94 °C for 45 s, primer annealing at 62 °C (*intI1* and *intI2*) or 60 °C (*intI3*) for 35 s, primer extension at 72 °C for 2 min, and a final elongation at 72 °C for 7 min. Positive and negative controls [17] were included in all PCR assays, and 1 kb ladder (Invitrogen) was used as a molecular size standard. After amplification, PCR products were separated by electrophoresis on 1% agarose gel in 1× TBE buffer, stained with ethidium bromide and visualized by UV transillumination. *E. coli* C828, *K. pneumoniae* C933 (provided both by Centro de Investigación Biomédica de la Rioja) and *E. coli* isolated from hospital inpatients, confirmed as carrying *intI2* by DNA sequencing, were used as positive controls for *intI1*, *intI3*, and *intI2*, respectively.

### 2.3. Detection of Insertion Sequences

DNA extracts were examined for the detection of different insertion sequences associated with ESBL genes, performing PCRs assays using the specific primers and conditions showed in Table 3 [27,32,33].

The PCRs were performed in a final volume of 25 µL containing 2 µL of DNA extract mixed with 2.5 µL of 10× buffer (Bioline, London, UK), 5 µL of dNTPs (Bioline, London, UK), 1.5 µL of MgCl_2_ 50 mM (Bioline, London, UK), 2 µL of each primer (Sigma-Aldrich, Madrid, Spain), and 1.5 U of Inmolase™ DNA polymerase (Bioline, London, UK), in a DNA thermal cycler GeneAmp^®^ PCR system 2700 (Applied Biosystems Division, Foster City, CA, USA). Amplification conditions were modified in order to improve the specificity using an initial denaturation at 94 °C for 12 min, followed by 35 cycles of DNA denaturation at 94 °C for 1 min, and primer annealing temperature depending on the IS (Table 3), primer extension at 72 °C for 2 min, and a final elongation at 72 °C for 10 min. PCR products were separated by electrophoresis on 1% agarose gels and were visualized under UV light after staining with ethidium bromide.

### 2.4. Sequence Analysis

Amplicons obtained in the different PCRs were sequenced to confirm the presence of integrons and insertion sequences. Bidirectional DNA sequence analysis was performed by the Macrogen EZ-Seq purification service (Macrogen Europe, Amsterdam, The Netherlands). Searches for DNA and protein homologies were carried out using the National Center for Biotechnology Information (http://www.ncbi.nlm.nih.gov/) using the BLAST program and the alignment of DNA and amino acids sequences were performed using Clustal Omega (http://www.ebi.ac.uk/Tools/msa/clustalo/).

### 2.5. Statistical Analysis

The results were subjected to statistical processing with the SPSS 15 software (SPSS Inc., Chicago, IL, USA), applying the chi-square test with a level of significance of *p* < 0.05.

## 3. Results and Discussion

### 3.1. Distribution of Integrons in ESBL-Producing E. coli

**Figure 1:**
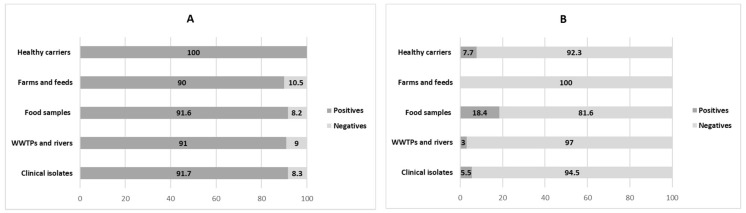
Prevalence (percentages) and distribution of (**A**)class 1 (*intI1*) and (**B**) class 2 (*intI2*) integrons in ESBL-producing *E. coli* according to their origin. ESBL: Extended spectrum beta-lactamases.

The occurrence and types of integrons, according to the isolate origin, is presented in Figure 1. As expected, class 1 showed the highest dissemination, being present in 92% of the isolates (*n* = 138) and in all environments, without significant differences among them (*p* < 0.05). Class 1 integrons have been reported as the most ubiquitous type among enteric bacteria [34,35,36]. In a similar way, Solberg et al. [37] reported the presence of class 1 integron in 70% of *E. coli* causing community-acquired infections. According to Roe et al. [38], the occurrence of class 1 integrons in healthy people suggests a possible acquisition of resistance genes circulating in different environments by a constant horizontal exchange of these genes. By contrast, class 2 integron was found in only 13 strains (8.5%), in accordance with the study of Ozgumus et al. [39], who found this class of integron in pathogenic, environmental, and commensal *E. coli* with a lower frequency than class 1. Finally, *intI3* was not detected, similarly to the report by Vinué et al. [40]. In fact, limited studies describe the presence of class 3 integron in *E. coli* [14,41] and, to date, there are no published data reporting the presence of this integron in ESBL-producing *E. coli* isolated from Spain.

It should be noted that *intI2* was mainly detected in food isolates (18.4%), but not in farming environments (*p* = 0.044). This situation seems a little bit contradictory, but it could be due to the low number of isolates coming from farms and feed (*n* = 20), compared with food (*n* = 48). Probably, if we extended the study by increasing *n*, we would find positive results for this type of integron, as shown in the literature. In any case, our results are comparable to those obtained by Goldstein et al. [42], who demonstrated the presence of class 1 and class 2 integrons in food, livestock, and water contaminated with farm animal feces. In a similar way, *intI2* has been detected in poultry products [38].

In addition, it is remarkable that *intI1* and *intI2* coexist in 8% of the isolates (92.1% of those carrying *intI2*). Rizk et al. [20] reported the co-existence of more than one type of integron in 36.9% of isolates, and a prevalence of 38% was reported by Kargar et al. [41] in a study performed in 69 multidrug-resistant (MDR) *E. coli*. By contrast, Kor et al. [43] found only one isolate carrying both integrons among clinical isolates, and Odetoyin et al. [16] reported a prevalence of 2.4% in fecal *E. coli* isolated from mother–child pairs in Nigeria. The simultaneous existence of multiple integrons represents a great threat for the dissemination of antimicrobial resistance genes among Enterobacteriaceae [31].

### 3.2. Analysis of Insertion Sequences

The prevalent type was IS*26* (99.4%), followed by IS*Ecp1* (68%) and IS*903* (65.3%), while IS*CR1* was detected only in 19 isolates (12.6%). The wide presence of IS26 in almost all multidrug-resistant isolates is a hint that IS26 is not associated with multidrug resistance, but only with ESBL-producing isolates.

**Figure 2:**
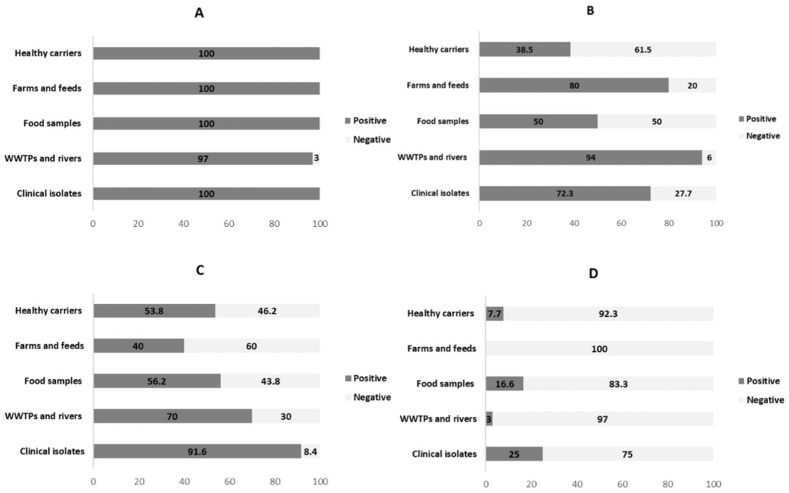
Prevalence (percentages) and distribution of insertion sequences in ESBL-producing *E. coli* according to their origin. (**A**) IS*26*; (**B**) IS*Ecp1*; (**C**) IS*903*; (**D**) IS*CR1*.

The four insertion sequences were present in all environments (Figure 2), except IS*CR1*, that was not detected in farm and feeds. This latest result contrasts with the reported by Ali et al. [44], that showed the connection between IS*CR1* and *intI1* in strains isolated from diverse dairy farms in China. However, the wide dissemination of IS among different niches has been reported by other authors. For instance, Cullik et al. [25] showed the association between *bla*_CTX-M_ with the common elements IS*Ecp1*, IS*26*, and IS*903*, in ESBL-producing *E. coli* isolated in a German Hospital. The IS*Ecp1* type has been detected in clinical isolates from Korea, and in isolates from healthy or diseased food-producing animals, including swine and avian [45,46].

**Figure 3:**
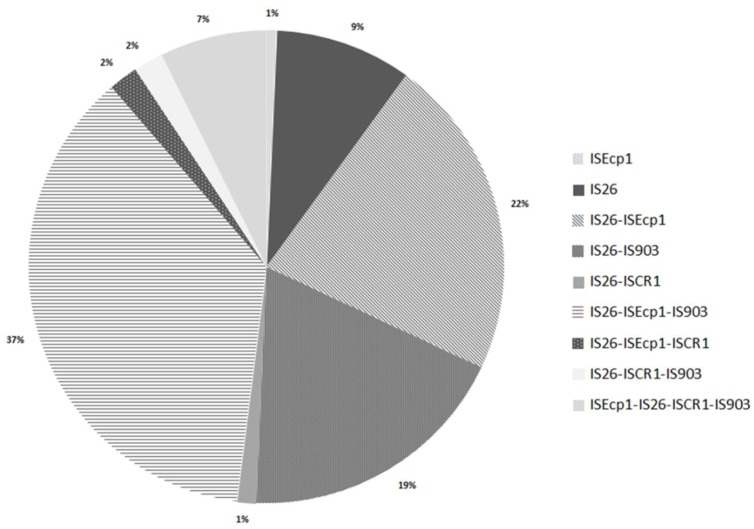
Genetic patterns and prevalences among the studied ESBL-producing *E. coli*.

In addition, the frequent co-existence of several insertion sequences in the same strain has been detected, in agreement with other studies [25,26,27,47]. Genetic patterns are presented in Figure 3, showing that the majority of isolates carried two or three IS (42% and 40.7%, respectively), whereas 10% of them carried only one. The three prevalent genetic patterns were IS*26*–IS*Ecp1*–IS*903* (*n* = 55), IS*26*–IS*Ecp1* (*n* = 33), and IS*26*–IS*903* (*n* = 28) (37%, 22%, and 19%, respectively). The combination IS*26*–IS*Ecp1* has been related with the pathogen clone ST131 [25,48], and it was present in 4 isolates coming from healthy people (*n* = 2) and hospital inpatients (*n* = 2), that supposes a possible risk situation for the healthy population. Finally, it is remarkable that 7.3% of isolates contain the four IS (IS*Ecp1*–IS*26*–IS*903*–IS*CR1*), a situation that, to our best knowledge, is being described in the literature for the first time. These isolates come mainly from hospital inpatients (*n* = 9), but we also found the genetic patterns in isolates from a river (*n* = 1) and from a chicken hamburger (*n* = 1). In summary, these results show the complexity of mobile genetic elements, and suggest the facility to acquire different mechanisms to disseminate resistance genes through all environments.

### 3.3. The Important Role of Horizontal Genetic Elements in the Dissemination of ESBLs

Correlation between the presence of genetic elements and ESBL has been reported by several authors [25,49,50], and our results support this fact (Table 4). IS*26* have been observed flanking the open reading frame (orf) regions of β-lactamase genes [51], and prevalences higher than 94% in all ESBL types were observed in this study. Similar results were detected in a study carried out in Kenya with 27 *E. coli* strains obtained from hospitalized patients, in which over 40% of isolates carrying *bla*_TEM-52_, *bla*_SHV-5_, or *bla*_CTX-M-14_, were linked to the IS*26* [50]. Otherwise, Billard-Pomares et al. [52] reported the characterization of a P1 bacteriophage from an ESBL-*E. coli* strain which had acquired two foreign DNA fragments, one of them being a fragment mobilized by two IS*26* elements containing a *bla*_SHV-2_ gene. Finally, Doi et al. [53] reported the relation between OXA (Beta lactamase product of blaOXA genes) and IS*26* downstream of a class 1 integron in a *K. pneumoniae* strain. In summary, as Cullik et al. affirm [25], IS*26* have an important role in the spread of resistance genes.

Similarly, IS*Ecp1*-like insertion sequences have been observed upstream of orfs encoding members belonging to the CTX-M-1, CTX-M-2, and CTX-M-9 clusters. Kim et al. [45] found the association of IS*Ecp1* and CTX-M in clinical isolates, especially in strains containing CTX-M-14 (in agreement with the 37% observed in our study). A similar association was found in China by Sun et al. [54] in healthy and sick pets. In addition, Tamang et al. [55] reported that 97.6% *bla*_CTX-M_ genes (isolated from cattle, farm workers, and the farm environment) possessed the insertion sequence IS*Ecp1* upstream of *bla*_CTX_. On the other hand, our results show that 9 out of 102 isolates carrying IS*Ecp1* (isolated from WWTP, river, farm soil and feed) were disrupted by IS*26*. Similar findings have been reported in a German University Hospital [25], where cases of IS*Ecp1* disrupted by an intact IS*26* were detected. In the same way, Wang et al. [48] detected a truncated copy of IS*Ecp1* gene with an IS*26* gene being located upstream in 3 out of 9 ESBL-producing *E. coli* isolated from fecal samples of food producing animals and healthy humans. Finally, despite the lower prevalence of IS*CR1* observed in the present study (12.6%), the aforementioned IS is another important element in the genetic platforms associated with the dissemination of CTX-M genes [22,56,57]. In general, IS*CR1* has been associated with CTX-M-2 and CTX-M-9 subtypes [57,58,59], but the majority of our strains carrying this IS were CTX-M-14 and CTX-M-15 producers. That could explain the low number of strains carrying IS*CR1*. Moreover, IS*CR1* mediates the formation of a complex with class 1 integrons [23,57]. From the total of isolates carrying IS*CR1*, 94.7% contain *intI1*, and even one of them contained both integrons (*intI1* and *intI2*). However, we have not found a specific association between the isolates containing *intI1* and the different ESBLs, due to its wide presence (92% of isolates). On the other hand, CTX-M-14 was present in the 46% of the isolates containing *intI2* (the same as SHV-12), whereas TEM and CTX-M-1 were detected in 38.5% and 15.4%, respectively, of *intI2* carriers.

Moreover, it must be pointed out that IS*Ecp1*-IS*903* is known as one of the major genetic platforms [22,27,54]. Our results showed that IS*903* and IS*Ecp1* were present in 55 isolates in co-existence with IS*26* (IS*26*-IS*Ecp1*-IS*903*). Similarly, a recent report detected this genetic platform [46] in CTX-M-14-producing *E. coli* isolated from animals [48]. Furthermore, all the analyzed strains show multidrug-resistant (MDR) phenotype, which means that they are resistant to at least three different classes of antimicrobials [28,29]. Similar results were reported by Woodford et al. [60] that found the plasmid pEK499 harboring 10 genes that confer resistance to eight antibiotic classes and also carrying IS (IS*26* and IS*Ecp1*).

Finally, Table 5 summarizes the relationship between the number of IS present in the same isolate, and the number of ESBL types produced by each microorganism. It can be seen that as the number of ESBL enclosed in the same genetic environment increases, the number of insertion sequences present also increases.

To sum up, the MDR ESBL-producing *E. coli* analyzed in the present study carried at least one genetic element (integron and IS). Since the strains were isolated from different sources (clinical isolates, healthy carriers, farms and feeds, food samples, WWTPs and rivers), these data revealed the potential risk for the dissemination of antimicrobial resistances among environmental and human bacteria.

## 4. Conclusions

In conclusion, this study highlights the high prevalence of different horizontal genetic elements among ESBL-producing *E. coli* isolates from food, environmental, and human samples. The analysis of integrons, showed that *intI1* was present in the majority of strains and in all sources, while the prevalence of *intI2* was lower but remarkable in the food isolates. Concerning insertion sequences, the multiple associations, like IS*26*-IS*Ecp1*, are relevant. Thus, the co-existence of diverse types of integrons and insertion sequences suggest possible risk for the dissemination of resistance genes among different environments and, therefore, additional investigations regarding the genetic composition of these integrons and insertion sequences are encouraged, to understand the role of these mobile elements in the spread of multidrug-resistant bacteria.

## Figures and Tables

**Table 1:** Genotypic characteristics of extended-spectrum β-lactamase (ESBL)-producing *E. coli* according to their origin [5,28,29,30].

Sample Origin	Percentages of Detected *bla* Genes					
	*bla*_CTX-M-14_	*bla*_CTX-M-15_	*bla*_CTX-M-1_	*bla*_TEM-42_	*bla*_TEML-171_	*bla*_SHV-12_
Hospital inpatients	41.7	61.1	8.3	11.1	NA	5.5
Healthy people	46.2	30.8	15.4	0	46.2	0
WWTP and rivers	33.3	30.3	18.2	6	NA	6
Food	32.7	4.1	18.3	12.3	31.8	35.6
Farms and feeds	31.6	5.26	47.4	26.3	5	21

NA: Not analyzed.

**Table 2:** Primers used for the detection of integrons.

Primer	Sequence (5′-3′)	Amplicon Size (pb)	T (°C ) ^3^	GenBank Accession No	Reference
*intI1*-Fw ^1^	GGTCAAGGATCTGGATTTCG	483	62	U49101	[31]
*intI1*-Rv ^2^	ACATGCGTGTAAATCATCGTC	483	62	U49101	[31]
*intI2*-Fw ^1^	CACGGATATGCGACAAAAAGGT	789	62	L10818	[31]
*intI2*-Rv ^2^	TAGCAAACGAGTGACGAAATG	789	62	L10818	[31]
*intI3*-Fw ^1^	AGTGGGTGGCGAATGAGTG	600	60	D50438	[31]
*intI3*-Rv ^2^	TGTTCTTGTATCGGCAGGTG	600	60	D50438	[31]

^1^ Fw: forward; ^2^ Rv: reverse; ^3^ T (°C): annealing temperature.

**Table 3:** Primers and conditions used for the amplification of insertion sequences.

Primer ^1^	Sequence (5′-3′)	Amplicon Size (pb)	T (°C) ^3^	GenBank Accession No.	Reference
IS*Ecp1*-Fw ^1^	ATCTAACATCAAATGCAGG	1381	60	AJ972954	[27]
IS*Ecp1*-Rv ^2^	AGACTGCTTCTCACACAT	1381	60	AJ972954	[27]
IS*26*-Fw ^1^	TCACTCCACGATTTACCGCT	557	61	AF205943	[27]
IS*26*-Rv ^2^	CTTACCAGGCGCATTTCGCC	557	61	AF205943	[27]
IS*CR1*-Fw ^1^	TCGCTGCGAGGATTGTCATC	1100	60	AF174129	[32]
IS*CR1*-Rv ^2^	CTCGCTTGAGGCGTTGCAT	1100	60	AF174129	[32]
IS*903*-Fw ^1^	CATATGAAATCATCTGCGC	473	56	EU056266	[33]
IS*903*-Rv ^2^	CCGTAGCGGGTTGTGTTTTC	473	56	EU056266	[33]

^1^ Fw: forward; ^2^ Rv: reverse; ^3^ T (°C): annealing temperature.

**Table 4:** Prevalences of insertion sequences and integrons among the different types of ESBL-*E. coli* producers.

*bla* Genes	IS*26*	IS*903*	IS*Ecp1*	IS*CR1*	*intI1*	*intI2*
*bla*_CTX-M_	99.2	90.3	79	11.3	94	6.5
*bla*_TEM_	100	89.9	88.5	16	94	7.3
*bla*_OXA-1_	94.5	83.3	50	5.5	100	0
*bla*_SHV_	100	56.5	26	0	95.7	21.8

**Table 5:** Relationship between number of IS in each isolate and the number of expressed ESBLs.

Number of IS in Each Isolate	N Isolates	N Isolates (%) Producing			
		1 ESBL	2 ESBL	3 ESBL	4 ESBL
1	15	46.6	40	0	0
2	63	46	46	8	0
3	61	49	41	8.2	1.6
4	11	0	81.8	18.2	0

## Supplementary Materials

Table S1: Antimicrobial profiles of ESBL-producing *E. coli* according to their origin; Table S2: Phenotypic and genotypic characteristics of strains isolated from hospital inpatients (*n* = 36); Table S3: Phenotypic and genotypic characteristics of aquatic strains included in the study (*n* = 33); Table S4: Phenotypic and genotypic characteristics of strains isolated from food (*n* = 48); Table S5: Phenotypic and genotypic characteristics of strains from farm origin included in the study (*n* = 20); Table S6: Phenotypic and genotypic characteristics of strains isolated from healthy people (*n* = 13).
