# Supplementary material for: Prevalence of Integrons and Insertion Sequences in ESBL-Producing E. coli Isolated from Different Sources in Navarra, Spain
Source: Int J Environ Res Public Health. 2018 Oct 20;15(10):2308. doi: 10.3390/ijerph15102308 (PMC6209886; doi:10.3390/ijerph15102308)
Supplement: Supplementary file 1 [file ijerph-15-02308-s001.pdf]

Supplementary Materials: Prevalence of Integrons and Insertion Sequences in ESBL-Producing *E. coli* Isolated from Different Sources in Navarra, Spain

Lara Pérez-Etayo [1,*], Melibea Berzosa [1], David González [1,2] and Ana Isabel Vitas [1,2]

[1] Department of Microbiology and Parasitology, University of Navarra, 31008 Pamplona, Spain; mberzosa.1@alumni.unav.es (M.B.); dgonzalez@unav.es (D.G.); avitas@unav.es (A.I.V.)
[2] IDISNA, Navarra Health Research Institute, 31008 Pamplona, Spain
* Correspondence: lperez.13@alumni.unav.es; Tel.: +34-948-425-600

Received: 14 September 2018; Accepted: date; Published: date

Table S1. Antimicrobial profiles of ESBL-producing *E. coli* according to their origin.

Family	Antibiotic[1]	Origin of Strains				
		Hospital inpatients	Healthy people	WWTPs and rivers	Food	Farms and Feeds
	AMP	100	92.3	100	100	100
Penicillin	PIP	100	76.9	100	100	100
	MZ	100	84.6	100	100	100
	CZ	100	92.3	100	100	100
	CXM	100	92.3	100	100	100
	CPD	100	92.3	100	100	100
Cephalosporin	CTX	100	84.6	100	100	100
	CAZ	100	92.3	97	100	95
	FOX	8.3	7.6	3	4.2	20
	FEP	97.2	84.6	97	100	100
Monobactams	AZT	100	84.6	97	100	100
	AMC	47.2	23	6	4.2	10
β-lactamase inhibitor	AMS	100	46.1	69	41.6	65
	TZP	25	0	0	2.1	0
	ETP	0	0	0	2.1	0
Carbapenem	MER	0	0	0	2.1	0
	IMP	0	0	0	2.1	0
	AK	2.7	0	0	0	0
Aminoglycosides	GM	44.5	7.6	24.2	8.3	10
	TO	61	7.6	18.2	4.1	10
	LV	69.5	53.8	27.3	39.5	65
Quinolones	CIP	69.5	7.6	33.3	60.4	70
	MXF	69.5	53.8	30.3	64.5	90
Tetracycline	TET	80.5	30.7	75.7	91.6	90

	TIG	0	0	0	2	0
	COL	22.2	15.4	30.3	6.2	15
	SXT	80.5	46.1	39.3	41.6	35
Others	FOT	8.3	7.6	0	2	25
	FM	0	0	0	8.3	0
	CHL	16.6	0	12.1	31.2	10

[1] Ampicillin, AMP; piperacillin, PIP; mezlocillin, MZ; cefazolin, CZ; cefuroxime, CXM; cefpodoxime, CPD; cefotaxime, CTX; ceftazidime, CAZ, cefoxitin, FOX; cefepime, FEP; aztreonam, AZT; amoxicillin clavulanic acid, AMC; amoxicillin sulbactam, AMS; piperacillin tazobactam, TZP; ertapenem, ETP; meropenem, MER; imipenem, IMP; Amikacin, AK; gentamicin, GM; tobramycin, TO; levofloxacin, LV; ciprofloxacin, CIP; moxifloxacin, MXF; tetracycline, TET; tigecycline, TIG; colistin, COL; trimethoprim sulfamethoxazole, SXT; fosfomycin, FOT; nitrofurantoin, FM; chloramphenicol, CHL.

Table S2. Phenotypic and genotypic characteristics of isolates from hospital inpatients (*n*=36)

Nº	β-lactamase genes	Phylogroup	MLST		Integrons		Insertion Sequence			
			Sequence type (ST)	Clonal complex (CC)	intI1	intI2	ISEcp1	IS26	IS903	ISCR1
1	*bla*$_{CTX-M-3}$, *bla*$_{OXA-1}$	B2	ST838	ST131	+	-	-	+	+	-
2	*bla*$_{CTX-M-14}$, *bla*$_{TEM-42}$	A	ST453	ST86	+	+	+	+	+	-
3	*bla*$_{CTX-M-1}$, *bla*$_{TEM-1}$	D	ST563	ST398	+	-	+	+	+	-
4	*bla*$_{CTX-M-15}$, *bla*$_{TEM-42}$	B2	ST3329	ST131	+	-	+	+	+	-
5	*bla*$_{CTX-M-14}$, *bla*$_{TEM-1}$	A	ST98	ST10	+	+	+	+	+	-
6	*bla*$_{CTX-M-15}$, *bla*$_{TEM-1}$	B2	ST3329	ST131	+	-	+	+	+	-
7	*bla*$_{CTX-M-15}$, *bla*$_{OXA-1}$	D	ST648	ST648	+	-	+	+	+	+
8	*bla*$_{CTX-M-15}$, *bla*$_{OXA-1}$	B2	ST3213	ST131	+	-	-	+	+	-
9	*bla*$_{CTX-M-1}$, *bla*$_{CTX-M-14}$, *bla*$_{TEM-42}$	D	ST867	ND	+	-	+	+	+	+
10	*bla*$_{CTX-M-14}$, *bla*$_{CTX-M-15}$, *bla*$_{TEM-1}$	B1	ST448	ST448	+	-	+	+	+	-
11	*bla*$_{CTX-M-15}$, *bla*$_{OXA-1}$	B2	ST3213	ST131	+	-	-	+	+	-
12	*bla*$_{CTX-M-15}$, *bla*$_{OXA-1}$	B2	ST2704	ST131	+	-	-	+	+	-
13	*bla*$_{CTX-M-15}$, *bla*$_{TEM-1}$, *bla*$_{OXA-1}$	D	ST405	ST405	+	-	-	+	+	-
14	*bla*$_{CTX-M-15}$, *bla*$_{OXA-1}$	B2	ST3878	ST131	+	-	-	+	+	-
15	*bla*$_{CTX-M-15}$, *bla*$_{TEM-1}$, *bla*$_{OXA-1}$	D	ST405	ST405	+	-	-	+	+	-
16	ND[1]	A	ST617	ST10	+	-	+	+	+	+
17	ND	D	ST648	ST648	+	-	-	+	-	-
18	*bla*$_{CTX-M-15}$, *bla*$_{OXA-1}$	D	ST72	ST405	+	-	-	+	+	-
19	*bla*$_{CTX-M-14}$, *bla*$_{OXA-1}$	B2	ST73	ST73	+	-	+	+	+	-
20	*bla*$_{CTX-M-15}$, *bla*$_{OXA-1}$	B2	ST3878	ST131	+	-	+	+	-	-
21	*bla*$_{CTX-M-14}$,*bla*$_{CTX-M-15}$, *bla*$_{TEM-1}$	A	ST617	ST10	+	-	+	+	+	-
22	*bla*$_{CTX-M-14}$, *bla*$_{CTX-M-15}$, *bla*$_{TEM-1}$	D	ST69	ST69	+	-	+	+	+	+
23	*bla*$_{CTX-M-15}$, *bla*$_{TEM-1}$	D	ST405	ST405	+	-	+	+	+	+
24	*bla*$_{CTX-M-14}$, *bla*$_{TEM-1}$	A	ST410	ST23	+	-	+	+	+	+
25	*bla*$_{CTX-M-15}$, *bla*$_{OXA-1}$	D	ST648	ST648	+	-	+	+	+	-
26	*bla*$_{CTX-M-15}$, *bla*$_{OXA-1}$	B1	ST94	ST448	+	-	-	+	+	-
27	*bla*$_{CTX-M-14}$	D	ST405	ST405	-	-	+	+	+	-
28	*bla*$_{CTX-M-14}$, *bla*$_{TEM-42}$	B2	ST95	ST95	+	-	+	+	+	-
29	*bla*$_{CTX-M-15}$, *bla*$_{TEM-1}$	D	ST393	ST31	+	-	+	+	+	+
30	*bla*$_{CTX-M-15}$, *bla*$_{TEM-1}$	B2	ST838	ST131	-	-	+	+	+	-
31	*bla*$_{CTX-M-1}$, *bla*$_{CTX-M-14}$	D	ST685	ND	+	-	+	+	+	+
32	*bla*$_{CTX-M-14}$, *bla*$_{CTX-M-15}$	D	ST4849	ND	+	-	+	+	+	+
33	*bla*$_{CTX-M-15}$, *bla*$_{TEM-42}$, *bla*$_{OXA-1}$	B2	ST4354	ST131	+	-	+	+	+	-
34	*bla*$_{CTX-M-14}$, *bla*$_{TEM-1}$	B2	ST3213	ST131	-	-	+	+	-	-
35	*bla*$_{CTX-M-14}$, *bla*$_{CTX-M-15}$, *bla*$_{TEM-1}$, *bla*$_{SHV-12}$	A	ST410	ST23	+	-	+	+	+	-
36	*bla*$_{CTX-M-14}$, *bla*$_{TEM-1}$	A	ST617	ST10	+	-	+	+	+	-

Table S3. Phenotypic and genotypic characteristics of aquatic isolates included in the study (*n*=33)

Nº	Source[1]	β-lactamase genes	Phylogroup	MLST: Sequence type (ST)	MLST: Clonal complex (CC)	Integrons: intI1	Integrons: intI2	Insertion Sequence: ISEcp1	Insertion Sequence: IS26	Insertion Sequence: IS903	Insertion Sequence: ISCR1
1	WWTP	$bla_{\text{CTX-M-14}}$	B1	ST616	ST155	+	-	+	+	-	-
2	WWTP	$bla_{\text{CTX-M-15}}$, $bla_{\text{OXA-1}}$	A	ST617	ST10	+	-	+	+	+	-
3	WWTP	$bla_{\text{CTX-M-15}}$	B2	ST3264	ND[2]	+	-	+	+	+	-
4	WWTP	$bla_{\text{CTX-M-1}}$	B1	ST906	ND	+	-	+	+	+	-
5	WWTP	$bla_{\text{CTX-M-15}}$, $bla_{\text{TEM-1}}$, $bla_{\text{OXA-1}}$	B1	ST4764	ND	+	-	+	+	+	-
6	WWTP	$bla_{\text{CTX-M-14}}$, $bla_{\text{TEM-1}}$	D	ST57	ST350	+	-	+	+	-	-
7	WWTP	$bla_{\text{CTX-M-15}}$	D	ST950	ND	+	-	+	+	-	-
8	WWTP	$bla_{\text{CTX-M-14}}$	A	ST615	ST46	+	-	+	+	+	-
9	WWTP	$bla_{\text{CTX-M-1}}$	A	ST93	ST168	+	-	+	+	+	-
10	WWTP	$bla_{\text{CTX-M-1}}$	D	ST69	ST69	+	-	+	+	-	-
11	WWTP	$bla_{\text{CTX-M-14}}$, $bla_{\text{TEM-1}}$	A	ST167	ST10	+	-	+	+	-	-
12	WWTP	$bla_{\text{CTX-M-1}}$	B1	ST162	ST469	+	-	+	+	+	-
13	WWTP	$bla_{\text{CTX-M-15}}$	A	ST615	ST46	-	-	+	+	+	-
14	WWTP	$bla_{\text{TEM-42}}$, $bla_{\text{SHV-12}}$	A	ST611	ST156	+	-	+	+	+	-
15	WWTP	$bla_{\text{CTX-M-14}}$	B1	ST446	ST446	+	-	+	+	+	-
16	WWTP	$bla_{\text{CTX-M-14}}$, $bla_{\text{TEM-1}}$	D	ST686	ND	+	-	+	+	+	-
17	R	$bla_{\text{CTX-M-1}}$, $bla_{\text{TEM-1}}$	D	ST906	ND	+	-	+	+	-	-
18	R	$bla_{\text{CTX-M-14}}$	D	ST3843	ND	+	-	+	+	+	-
19	R	$bla_{\text{CTX-M-15}}$	D	ST949	ND	+	-	+	+	-	-
20	WWTP	$bla_{\text{CTX-M-14}}$	D	ST398	ST398	+	-	+	+	+	-
21	WWTP	$bla_{\text{CTX-M-15}}$, $bla_{\text{OXA-1}}$	D	ST4764	ND	-	-	+	-	-	-
22	WWTP	$bla_{\text{CTX-M-1}}$	D	ST469	ST469	+	-	+	+	+	-
23	WWTP	$bla_{\text{CTX-M-55}}$	A	ST3065	ND	+	-	+	+	+	-
24	R	$bla_{\text{CTX-M-1}}$, $bla_{\text{TEM-1}}$	B1	ST1285	ND	+	-	+	+	+	-
25	R	$bla_{\text{CTX-M-1}}$	D	ST315	ST38	+	-	+	+	+	-
26	R	$bla_{\text{CTX-M-1}}$, $bla_{\text{TEM-42}}$	A	ST98	ST10	+	-	+	+	+	-
27	R	$bla_{\text{CTX-M-15}}$, $bla_{\text{TEM-1}}$	B1	ST4212	ND	+	-	-	+	+	-
28	R	$bla_{\text{CTX-M-15}}$, $bla_{\text{TEM-1}}$	A	ST3530	ND	+	-	+	+	-	-
29	R	$bla_{\text{SHV-12}}$	B1	ST1196	ND	-	+	-	+	+	-
30	R	$bla_{\text{CTX-M-15}}$, $bla_{\text{TEM-1}}$	A	ST4972	ND	+	-	+	+	+	+
31	R	$bla_{\text{CTX-M-14}}$	D	ST915	ND	+	-	+	+	+	-
32	R	$bla_{\text{CTX-M-14}}$	A	ST615	ST46	+	-	+	+	+	-
33	R	$bla_{\text{CTX-M-14}}$	A	ST615	ST46	+	-	+	+	-	-

.[1] R: river; WWTP: waste water treatment plant [2] ND: Not detected

Table S4. Phenotypic and genotypic characteristics of isolates from food (*n*=48).

Nº	Source[1]	β-lactamase genes	Phylogroup	MLST		Integrons		Insertion Sequence			
				Sequence type (ST)	Clonal complex (CC)	intI1	intI2	ISEcp1	IS26	IS903	ISCR1
1	C	*bla*$_{CTX-M-14}$	D	ST648	ST648	+	+	+	+	+	-
2	P	*bla*$_{CTX-M-14}$	A	ST615	ST46	-	-	-	+	+	-
3	C	*bla*$_{CTX-M-14}$, *bla*$_{TEM-1}$	A	ST615	ST46	+	-	+	+	+	-
4	C	*bla*$_{CTX-M-1}$	B1	ST155	ST155	+	-	+	+	+	-
5	C	*bla*$_{CTX-M-1}$	B2	ST604	ST101	+	-	+	+	+	-
6	P	*bla*$_{CTX-M-1}$	B1	ST179	ST155	+	-	+	+	-	-
7	P	*bla*$_{TEM-42}$	B1	ST776	ND	-	-	-	+	+	-
8	C	*bla*$_{TEM-1}$, *bla*$_{SHV-12}$	D	ST880	ND	+	-	-	+	-	-
9	B	*bla*$_{CTX-M-14}$, *bla*$_{TEM-42}$	B2	ST883	ND	+	-	+	+	-	-
10	B	*bla*$_{CTX-M-14}$	B1	ST776	ND	+	+	+	+	-	-
11	B	*bla*$_{CTX-M-1}$	D	ST880	ND	+	-	+	+	+	-
12	B	*bla*$_{CTX-M-14}$	D	ST57	ST350	+	+	+	+	-	+
13	C	*bla*$_{CTX-M-14}$	D	ST349	ST349	+	-	+	+	-	+
14	B	*bla*$_{CTX-M-14}$	B1	ST448	ST448	+	-	+	+	+	-
15	B	*bla*$_{TEM-55}$	B1	ST179	ST155	+	-	-	+	+	-
16	B	*bla*$_{CTX-M-1}$	A	ST373	ST168	+	-	+	+	-	-
17	P	*bla*$_{CTX-M-14}$, *bla*$_{TEM-1}$	A	ST44	ST10	+	-	-	+	+	-
18	C	*bla*$_{CTX-M-14}$	B2	ST619	ST101	+	+	+	+	-	-
19	P	*bla*$_{CTX-M-2}$	D	ST955	ND	+	-	-	+	-	+
20	P	*bla*$_{CTX-M-14}$, *bla*$_{TEM-42}$	D	ST648	ST648	+	-	+	+	-	+
21	P	*bla*$_{CTX-M-14}$	A	ST453	ST86	-	-	+	+	+	-
22	C	*bla*$_{CTX-M-15}$, *bla*$_{TEM-42}$	A	ST43	ST10	+	-	+	+	+	+
23	C	*bla*$_{CTX-M-15}$, *bla*$_{TEM-42}$	B1	ST179	ST155	+	-	-	+	-	+
24	C	*bla*$_{CTX-M-14}$	A	ST4402	ND	+	-	+	+	+	-
25	C	*bla*$_{CTX-M-14}$	A	ST98	ST10	+	-	+	+	-	-
26	B	*bla*$_{CTX-M-8}$, *bla*$_{TEM-1}$	B1	ST949	ND	+	-	-	+	-	-
27	C	*bla*$_{CTX-M-2}$, *bla*$_{TEM-42}$	D	ST844	ND	+	-	-	+	-	-
28	C	*bla*$_{SHV-12}$	A	ST6648	ND	+	-	-	+	-	-
29	C	*bla*$_{CTX-M-1}$	F	ST117	ND	+	-	+	+	-	-
30	C	*bla*$_{TEML-171}$, *bla*$_{SHV-12}$	B1	ST101	ST101	+	-	-	+	-	-
31	C	*bla*$_{CTX-M-1}$	C	ST23	ST23	+	-	+	+	+	-
32	C	*bla*$_{SHV-12}$	F	ST2085	ND	+	-	-	+	-	-
33	C	*bla*$_{TEML-278}$, *bla*$_{SHV-12}$	ND	ST2599	ND	+	-	-	+	+	-
34	C	*bla*$_{CTX-M-1}$	A	ST373	ST168	+	-	+	+	+	-
35	C	*bla*$_{TEML-171}$, *bla*$_{SHV-12}$	ND	ST2062	ND	+	+	-	+	+	-
36	C	*bla*$_{SHV-12}$	F	ST354	ST354	+	+	-	+	+	-
37	C	*bla*$_{SHV-12}$	Clade I or II	ST23	ST 23	+	-	-	+	-	-
38	C	*bla*$_{SHV-12}$	A	ND	ND	+	-	-	+	-	-
39	C	*bla*$_{SHV-12}$	A	ST665	ND	+	+	-	+	-	-
40	C	*bla*$_{TEML-171}$, *bla*$_{SHV-12}$	F	ST354	ST35	+	-	-	+	+	-
41	C	*bla*$_{CTX-M-1}$, *bla*$_{SHV-12}$	A	ST93	ST168	+	+	+	+	+	-
42	C	*bla*$_{TEML-171}$, *bla*$_{SHV-12}$	B1	ST937	ND	+	-	-	+	+	-
43	C	*bla*$_{TEML-171}$, *bla*$_{SHV-12}$	A	ST10	ST10	+	+	+	+	+	-
44	C	*bla*$_{SHV-12}$	F	ST117	ND	+	-	-	+	+	-
45	C	ND[2]	F	ST117	ND	-	-	-	+	+	+
46	C	ND	B1	ST1524	ND	+	-	-	+	+	+

47	C	*bla*$_{\text{CTX-M-14}}$, *bla*$_{\text{TEML-171}}$, *bla*$_{\text{SHV-12}}$	A	ST6094	ND	+	-	-	+	+	-
48	C	ND	B1	ND	ND	+	-	+	+	+	-

[1] B: beef; P: pork; C: chicken [2] ND: Not detected

Table S5. Phenotypic and genotypic characteristics of isolates from farm origin included in the study (*n*=20)

Nº	Source[1]	β-lactamase genes	Phylogroup	MLST		Integrons		Insertion Sequence			
				Sequence type (ST)	Clonal complex (CC)	intI1	intI2	ISEcp1	IS26	IS903	ISCR1
1	S	*bla*$_{\text{TEM-1}}$, *bla*$_{\text{SHV-12}}$	B2	ST359	ND	-	-	-	+	-	-
2	S	*bla*$_{\text{CTX-M-}}$1, *bla*$_{\text{TEM-1}}$	B2	ST3333	ND	+	-	+	+	+	-
3	S	*bla*$_{\text{CTX-M-14}}$, *bla*$_{\text{TEM-42}}$	A	ST10	ST10	+	-	+	+	+	-
4	S	*bla*$_{\text{CTX-M-14}}$, *bla*$_{\text{TEM-42}}$	A	ST10	ST10	+	-	+	+	+	-
5	S	*bla*$_{\text{CTX-M-1}}$, *bla*$_{\text{TEM-1}}$	A	ST88	ST23	+	-	+	+	-	-
6	S	*bla*$_{\text{CTX-M-1}}$, *bla*$_{\text{TEM-42}}$	A	ST88	ST23	+	-	+	+	+	-
7	S	*bla*$_{\text{CTX-M-14}}$, *bla*$_{\text{TEM-1}}$	B2	ST610	ND	+	-	+	+	-	-
8	S	*bla*$_{\text{CTX-M-1}}$, *bla*$_{\text{TEM-1}}$, *bla*$_{\text{SHV-12}}$, *bla*$_{\text{OXA-1}}$	B2	ST891	ND	+	-	+	+	-	-
9	S	*bla*$_{\text{SHV-12}}$	B2	ST878	ND	-	-	-	+	-	-
10	S	*bla*$_{\text{CTX-M-14}}$	B2	ST883	ND	+	-	+	+	-	-
11	S	*bla*$_{\text{CTX-M-14}}$	B2	ST883	ND	+	-	+	+	-	-
12	S	*bla*$_{\text{CTX-M-15}}$, *bla*$_{\text{TEM-42}}$	A	ST617	ST10	+	-	-	+	+	-
13	S	*bla*$_{\text{CTX-M-1}}$	B2	ST619	ST101	+	-	+	+	-	-
14	S	*bla*$_{\text{CTX-M-1}}$, *bla*$_{\text{TEM-42}}$, *bla*$_{\text{SHV-12}}$	A	ST373	ST168	+	-	+	+	+	-
15	F	*bla*$_{\text{CTX-M-1}}$	B1	ST3561	ND	+	-	+	+	-	-
16	F	*bla*$_{\text{CTX-M-1}}$	B1	ST602	ST446	+	-	+	+	-	-
17	F	*bla*$_{\text{CTX-M-1}}$	B1	ST446	ST446	+	-	+	+	+	-
18	F	*bla*$_{\text{CTX-M-1}}$	B1	ST446	ST446	+	-	+	+	-	-
19	F	*bla*$_{\text{CTX-M-14}}$	A	ST88	ST23	+	-	+	+	-	-
20	F	*bla*$_{\text{CTX-M-14}}$, *bla*$_{\text{TEML-171}}$	C	ND[2]	ND	+	-	-	+	+	-

[1] S: farm soil; F: feed [2] ND: Not detected

Table S6. Phenotypic and genotypic characteristics of isolates from healthy people (*n*=13)

Nº	Sex[1]/age range	β-lactamase genes	Phylogroup	MLST		Integrons		Insertion Sequence			
				Sequence type (ST)	Clonal complex (CC)	intI1	intI2	ISEcp1	IS26	IS903	ISCR1
1	M$_{40-65}$	*bla*$_{\text{CTX-M-14}}$, *bla*$_{\text{TEM-116}}$	B1	ST971	ND	+	-	-	+	+	-
2	F$_{5-18}$	*bla*$_{\text{CTX-M-14}}$, *bla*$_{\text{TEM-116}}$	A	ST2404	ND	+	-	-	+	+	+
3	F$_{18-40}$	*bla*$_{\text{CTX-M-1}}$, *bla*$_{\text{TEML-171}}$	D	ST38	ST38	+	-	-	+	+	-
4	F$_{18-40}$	*bla*$_{\text{CTX-M-1}}$, *bla*$_{\text{TEML-171}}$	ND	ND	ND	+	+	-	+	+	-
5	M$_{18-40}$	*bla*$_{\text{CTX-M-14}}$, *bla*$_{\text{CTX-M-15}}$, *bla*$_{\text{TEML-171}}$	B2	ST131	ST13	+	-	+	+	-	-
6	F$_{18-40}$	*bla*$_{\text{CTX-M-14}}$	A	ND	ND	+	-	+	+	-	-
7	F$_{18-40}$	*bla*$_{\text{TEML-171}}$	A	ND	ND	+	-	+	+	-	-
8	M$_{40-65}$	*bla*$_{\text{CTX-M-15}}$, *bla*$_{\text{TEML-171}}$	B2	ST131	ST131	+	-	+	+	-	-
9	M$_{40-65}$	*bla*$_{\text{CTX-M-15}}$, *bla*$_{\text{TEML-171}}$	B2	ST131	ST131	+	-	+	+	+	-
10	M$_{>65}$	*bla*$_{\text{CTX-M-14}}$	B2	ST131	ST131	+	-	-	+	+	-
11	M$_{5-18}$	*bla*$_{\text{CTX-M-14}}$	B2	ST3483	ST131	+	-	-	+	+	-
12	F$_{5-18}$	*bla*$_{\text{CTX-M-15}}$	C	ND	ND	+	-	-	+	-	-
13	F$_{>65}$	ND[2]	D	ND	ND	+	-	-	+	-	-

[1] F: Female; M: Male [2] ND: Not detected
